# “Doing well”: Intraoperative entrustable professional activity assessments provided limited technical feedback

**DOI:** 10.1016/j.sopen.2024.02.008

**Published:** 2024-02-24

**Authors:** Riley Brian, Natalie Rodriguez, Connie J. Zhou, Megan Casey, Rosa V. Mora, Katherine Miclau, Vivian Kwok, Liane S. Feldman, Adnan Alseidi

**Affiliations:** a Department of Surgery, University of California San Francisco, San Francisco, CA, USA; b School of Medicine, University of California San Francisco, San Francisco, CA, USA; c Department of Surgery, McGill University, Montreal, Quebec, Canada

**Keywords:** Entrustable professional activities, Graduate medical education, Surgical assessment

## Abstract

**Background:**

Entrustable Professional Activities (EPAs) allow for the assessment of specific, observable, essential tasks in medical education. Since being developed in non-surgical fields, EPA assessments have been implemented in surgery to explore intraoperative entrustment. However, assessment burden is a significant problem for faculty, and it is unknown whether EPA assessments enable formative technical feedback. EPAs' formative utility could inform how surgical programs facilitate technical feedback for trainees. We aimed to assess the extent to which narrative comments provided through the Fellowship Council (FC) EPA assessments contained technical feedback.

**Methods:**

The FC previously collected EPA assessments for subspecialty surgical fellows from September 2020 to October 2022. Two raters reviewed assessments' narrative comments for inclusion of each skill area that makes up part of the Objective Structured Assessment of Technical Skills (OSATS). A third rater reconciled discrepant ratings.

**Results:**

During the study period, there were 3302 completed EPA assessments, including 1191 fellow self-assessments, 1124 faculty assessments, and 987 assessments without an identified assessor role. We found that assessments' narrative comments related to a median of two of the seven OSATS areas (IQR:1–2). There were no comments relevant to any of the seven OSATS areas in 16.0 % of all assessments.

**Conclusions:**

In this review of narrative comments for EPA assessments from the FC, we found that limited technical feedback of the kind included in the OSATS was provided in many assessments. These results suggest benefit to adjusting the EPA form, enhancing faculty development, or continuing additional types of targeted technical assessment intraoperatively.

**Key message:**

This analysis of narrative comments from fellowship EPA assessments showed that many assessments included limited technical feedback. To allow for continued technical feedback for fellows, these results highlight the need for further refinements of the EPA assessment form, additional faculty development, or ongoing use of other types of technical assessment.

## Highlights

• Entrustable professional activities (EPAs) are now used for assessment in surgery.
• We reviewed narrative comments from fellowship intraoperative EPA assessments.
• Assessors provided limited technical feedback in many EPA assessments.
• EPA assessment forms may need adjustment or require further faculty development.

## Introduction

Work in competency-based medical education (CBME) has highlighted the importance of frequent feedback and comprehensive assessment to promote skill development and allow for appropriate trainee progression [[1], [2], [3]]. To be done properly, the frequent feedback and assessment needed for CBME require a substantial investment of faculty time [4,5]. However, assessment burden is a significant problem for faculty and trainees that has been identified with the adoption of CBME [[5], [6], [7]]. Indeed, given the many demands on faculty members' time, it is unreasonable to continue to introduce required assessments without removing administrative burden in another form.

Assessments framed through Entrustable Professional Activities (EPAs) were first introduced in 2005 by ten Cate as a means to “decide when a trainee may be trusted to bear responsibility to perform a professional activity.” [8] Since that time, EPAs have gained enormous traction in CBME given their usefulness in integrating domains of performance [9]. They describe specific, observable, essential tasks with actionable consequences that can result from their assessment [10]. EPAs have been piloted in undergraduate and graduate medical education, with substantial recent work expanding EPAs into surgical fields [[11], [12], [13], [14], [15]]. EPAs in surgical training have explored the entrustment of intraoperative care to trainees across multiple sites and surgical subspecialties [16,17]. EPA implementation has been predicated on the completion of numerous EPA assessments [18]. While a study in radiology found the average time to complete an EPA assessment to be just over 3 min, EPA assessment forms vary in format and faculty have voiced concerns that administrative burden only detracts from time spent teaching [19,20].

Given that intraoperative EPA assessments may comment on technical skills related to a trainee's operative ability, it has been proposed that EPA assessments could supplant other established methods of intraoperative feedback, and thus reduce overall assessment burden [21]. Established methods of intraoperative technical feedback are numerous and varied. These include Operative Performance Rating Systems (OPRS), the Global Operative Assessment of Laparoscopic Skills (GOALS), the Global Evaluative Assessment of Robotic Skills (GEARS), and innumerable procedure-specific tools [[22], [23], [24], [25]]. Perhaps the most commonly known and used form of intraoperative technical feedback is the Objective Structured Assessment of Technical Skills (OSATS) [26]. Originally described in 1997, the OSATS has been extensively studied and there is strong validity evidence for its use in formative feedback [27]. The OSATS consists of a global rating scale in seven areas: respect for tissue, time and motion, instrument handling, knowledge of instruments, use of assistants, flow of operation and forward planning, and knowledge of specific procedure [26].

EPA assessment forms contain constructed response items to allow for narrative descriptions of trainees' technical performance [18]. Prior authors have emphasized the importance of assessors being specific in this narrative feedback [28,29]. If narrative comments in intraoperative EPA assessment forms provide formative technical feedback, this would suggest that other intraoperative assessment could be reduced, thus decreasing assessment burden. However, it is unknown how narrative comments from actual EPA assessments compare to other forms of formative technical feedback, such as the OSATS, with regard to the amount of technical detail included in their intraoperative feedback. Knowing EPA assessments' formative technical utility could help programs facilitate technical feedback for trainees and inform decisions around how best to synergize assessment tools. As such, the objective of this study was to assess the extent to which feedback provided through narrative comments in intraoperative EPA assessments was technical in nature.

## Methods

### Setting and participants

The Fellowship Council (FC), which oversees fellowship training programs in a number of surgical subspecialties, collected intraoperative EPA assessments from September 2020 to October 2022. The FC collected these assessments online for subspecialty surgical fellows from 107 programs representing diverse institutions with wide-ranging practice and assessment patterns. Fellows represented abdominal wall, bariatrics, flexible endoscopy, foregut, and hepatopancreatobiliary subspecialties. Following collection, the FC de-identified EPA assessments and provided them to investigators for this study. After a case, a fellow, faculty member, or both could complete an intraoperative EPA assessment. Assessment forms solicited an entrustment level from zero to four and then contained two constructed response items with regard to intraoperative performance: “Please explain why you chose this level, commenting on something the fellow does well” and “Please explain why you chose this level, commenting on something the fellow needs to improve.” Of note, the EPAs and the assessment forms used by the FC differed from EPAs and assessment forms in use by other groups. Faculty and fellows received training on CBME, EPAs, and completing the EPA assessment forms during a webinar. Additional details on data collection by the FC, including images of the assessment form, can be found in previously published work [18].

### Outcomes measured

Two raters from the author group read all narrative comments from constructed response items for intraoperative EPA assessments. Raters reviewed these narrative comments to determine each component of the OSATS on which there was feedback. For example, a comment stating, “Created holes during peritoneal flap dissection due to excessive force… improve gentle manipulation of tissue using robotic instruments” was noted to relate to Respect for Tissues based on its mention of tissue manipulation (Table 1). As decided during rater training before beginning the ratings, we considered positive, negative, and neutral notes about each area to be relevant comments if they pertained to an area of the OSATS. Raters discussed examples relating to each OSATS area to determine the range of relevant comments. We chose to use the OSATS as a reference given its wide use and strong formative validity evidence for providing technical feedback [26,27]. Unlike other methods of reviewing feedback, the OSATS focuses on technical aspects of performance [[30], [31], [32]]. We expected that EPA assessments describing intraoperative entrustment would generally contain feedback captured by the OSATS.

**Table 1:** Examples of comments relating to each of the Objective Structured Assessment of Technical Skills (OSATS) areas.

OSATS area	Example feedback comment
Respect for tissue	“Created holes during peritoneal flap dissection due to excessive force… improve gentle manipulation of tissue using robotic instruments”
Time and motion	“Improving efficiency such that there is no wasted time in the room. For instance, while the abdomen is inflating, the liver retractor holder can be placed”
Instrument handling	“Continue to work on using the full length of the stapler and adjusting [it] with slow and efficient movements”
Knowledge of instruments	“She should review alternative coagulation and hemostasis techniques and familiarize herself with the adjunct tools necessary to use them”
Use of assistants	“Work on retracting to better visualize the anatomy and to better direct… assistant for better exposure especially up by the spleen/fundus”
Flow of operation and forward planning	“Able to slow down during complex maneuvers and challenging dissection, while also expediting more straightforward portions of procedure”
Knowledge of specific procedure	“Has an excellent understanding of the flow of the case… Regarding the JJ – should watch videos, particularly paying attention to the small details that make a case ‘look easy,’ and then try to implement these”

Following independent rating determinations, the two raters met with a third rater to discuss issues that had arisen during rating assignment. The raters removed assessments with obvious submission errors from the dataset, such as multiple identical assessments describing unique events. A third rater then reconciled all inter-rater discrepancies. During comment review, raters analyzed the content of narratives and collated those of particular relevance to the original descriptions of each of the OSATS areas [26]. Raters also listed narratives relevant to no OSATS area and narratives that commented on entrustment but not technical skill. We reviewed and aggregated these comments to identify examples that could provide context to the ratings.

### Analysis of the outcomes

We generated descriptive data about the assessors, assessment areas, assigned entrustment levels, length of narrative comments in submitted assessments, and OSATS areas. We calculated Cohen's kappa with regard to inter-rater agreement using the formula as described by McHugh [33]. We compared ordinal data between fellow self-assessments and faculty assessments using a Wilcoxon rank-sum test after confirming that the distributions were not normal. We set statistical significance at *p* < 0.05. We performed analyses in Stata/IC 16.1 for Mac (Stata Corp, College Station, Texas).

## Results

### EPA assessment data

There were 3302 intraoperative EPA assessments completed during the study period, including 1191 fellow self-assessments, 1124 faculty assessments, and 987 assessments without an identified assessor role (Table 2). In order of declining frequency, assessments were completed for five main areas: bariatric, foregut, abdominal wall, hepatopancreatobiliary, and flexible endoscopy. There were 218 fellows for whom assessments were submitted, with a median of six assessments per fellow (IQR: 2–19). Assessments were completed for cases involving 296 faculty members, with a median of six assessments completed by each faculty member (IQR: 2–14). Fellows represented 107 programs. All entrustment levels were assigned, including Level 0 (trusted to observe only – *n* = 10; 0.3 %), Level 1 (trusted with direct supervision – *n* = 280; 8.5 %), Level 2 (trusted with indirect supervision for simple cases – *n* = 987; 29.9 %), Level 3 (trusted with indirect supervision for complex cases – *n* = 1379; 41.8 %), and Level 4 (trusted to execute without supervision but with availability for clinical oversight as needed – *n* = 646; 19.6 %). The median entrustment level selected in assessments was Level 3 (IQR: 2–3).

**Table 2:** Descriptive data for Entrustable Professional Activity (EPA) assessments completed through the Fellowship Council.

Total EPA assessments - n	3302
Total fellows assessed - n	218
Total involved faculty - n	296
Total involved programs - n	107

Assessor - n (%)	
Faculty	1124 (34.0)
Fellow	1191 (36.1)
Unidentified	987 (29.9)

EPA category - n (%)	
Abdominal wall	704 (21.3)
Bariatric	1367 (41.4)
Flexible endoscopy	214 (6.5)
Foregut	745 (22.6)
Hepatopancreatobiliary	272 (8.3)

The median number of combined words from the narrative comments of the two constructed response items was 22 per assessment (IQR: 13–36). Among the assessments, 18.5 % of the forms' narrative comments contained 10 or fewer words. Narrative comments were slightly longer when completed by faculty (median 22 words) compared to fellows (median 19 words) (*p* < 0.001).

### OSATS comparison

**Fig. 1:**
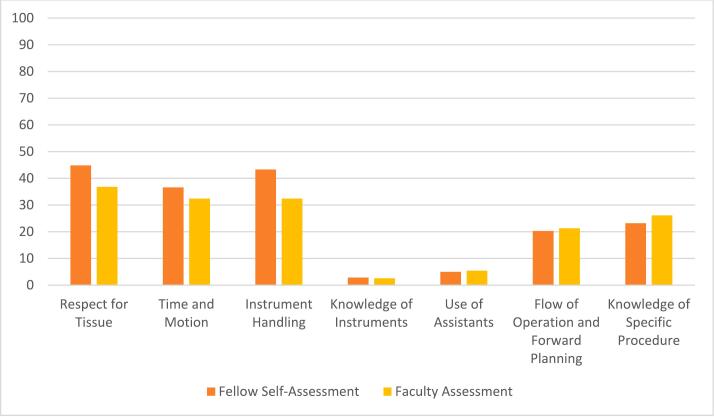
Percentage of Entrustable Professional Activity (EPA) assessments that contained comments relevant to each Objective Structured Assessment of Technical Skills (OSATS) area.

Two raters from our group each assigned 23,114 component OSATS ratings to the narrative comments from the EPA assessments. Of these, 21,024 (91.0 %) ratings were concordant, representing a kappa of 0.82. A third rater reconciled all discrepant ratings. Narrative comments in self-assessments from fellows and assessments from faculty both related to a median of two of the seven OSATS areas, though fellow comments ranked higher with regard to the number of OSATS areas discussed (*p* < 0.001). The most common OSATS area commented about was Respect for Tissue (44.5 %) while the least common OSATS area commented about was Knowledge of Instruments (2.5 %) (Fig. 1). Notably, narrative comments in 529 intraoperative assessments (16.0 %) contained no content relevant to any of the seven OSATS areas. Such narrative comments were generally brief or nonspecific as described below.

### Comment review

We collated examples of narrative comments to provide context to the quantitative findings. There was a wide range in the feedback provided in different assessments. Some assessments provided feedback without providing any technical feedback, with generic comments including “doing well,” “good,” “excellent,” “be better,” and “need to see more cases.” Other responses did not contain feedback at all, with narrative comments such as “complex patient,” “unusual case,” and “Whipple with vein involvement.” Some comments justified EPA level selection as not related to intraoperative technical ability, such as “would have been at a level 4, but this was our first lap ventral together.” Finally, we noted that some comments that did contain technical feedback pertaining to one of the OSATS areas were nonetheless not formative. For example, one comment simply stated “respect for tissues” without further notes; this comment does relate to one of the OSATS areas but was neither specific nor actionable. At the opposite end of the spectrum, a smaller number of comments contained thoughtful formative feedback. Specifically, one faculty member wrote:

[The fellow] knows the key steps of the operation in sequence. Tissue handling is overall safe. Getting better with use of the assistant to help her gain exposure. She is capable with robotic suturing and slip knot. Minor steps of the operation needed some prompting (e.g., removing fat pad/ hernia sac before mediastinal dissection). Be wary of dissecting on the esophageal muscle during reduction of the hernia sac. Overall use of the 3^rd^ arm was good, but can use it even more actively to get exposure to the mediastinum

This comment highlighted the ability of faculty members to provide actionable, formative feedback through the narrative comments.

## Discussion

EPAs support the implementation of CBME by providing a clear understanding of expected performance in a way that can be used for training and assessment – including intraoperatively. However, like all types of assessment, EPA assessments require time to be completed. Some have posited that this assessment burden can be alleviated by reducing existing technical assessment mechanisms, such as the quarterly standardized intraoperative assessments now required by the FC as an accreditation standard. Nonetheless, in our review of 3302 EPA assessments from the FC, we found that limited technical feedback was provided in many narrative comments for intraoperative assessments completed by both faculty assessors and fellow self-assessors.

Barriers resulting in limited technical feedback could include lack of prompting to provide technical feedback in the existing EPA assessment form, limited time to fill out the assessment form, or a view of the form as redundant to verbal feedback. There are multiple possible solutions to address these problems. First, the wording or items in intraoperative assessment forms may simply need to be adjusted. The current form asks for comments on something that the fellow did well and something that could be improved, but does not include any more specific questions. Pilot alterations could be tested with faculty members and fellows to assess for response process [34]. It is possible that small modifications could prompt more specific and effective feedback on assessments and thus allow for more technical feedback. Other specialties have used a wide variety of different EPA assessment forms and methods [19,35,36]. Second, additional emphasis likely needs to be placed on faculty development when EPA assessment forms are introduced. Faculty development has been proposed as central to the implementation of CBME, including for the assessment of EPAs [37,38]. Prior authors have highlighted that EPA assessment is about much more than the entrustment level assigned [28]. If this were emphasized to faculty members, they may be more inclined to provide the written formative feedback needed for trainees to improve. It was quite clear from our review of assessment comments that some faculty members invested significant thought into their feedback; these faculty members may serve as an example for training other faculty in assessment completion. Finally, though unlikely, it could be that EPA assessments are inadequate for technical feedback even though technical skill is necessary to be entrusted with performing a procedure. In that scenario, existing technical feedback mechanisms would need to continue and further discussion would center on reducing administrative or assessment burden in other areas. Interestingly, an interview-based study of 22 anesthesia residents and faculty concluded that EPA assessments were perceived to shift focus from nontechnical to technical skills [39]. This finding, though limited in its generalizability, suggests that EPA assessments do have the potential to facilitate technical feedback in a procedural setting. There are, of course, key differences between procedural and operative feedback. Additional work to investigate perceptions around technical feedback and EPA assessments among surgical faculty is ongoing by our group (unpublished data).

This study has multiple limitations. Although many institutions were involved, it reflects the narrative comments of EPA assessments submitted only for subspecialty fellows, and findings are likely not applicable to EPA assessments for surgical residents or other trainees. Indeed, given the quite high level of entrustment assigned on average, those completing the assessments may have felt it less relevant to provide feedback related to the type of fundamental technical skills described by the OSATS. Other authors have found differences in CBME assessment across the continuum of medical education, highlighting the need to investigate EPAs at all levels [40]. Furthermore, different institutions included in this study operationalize assessment in different ways and may not use, plan to use, or desire to use intraoperative EPAs for technical assessment. Similarly, the actual assessment form from the FC differs from other EPA assessment forms and this further limits generalizability [18]. Finally, the OSATS may not be the appropriate gold standard for technical feedback in these EPA assessment forms, and our study addressed quantity of OSATS-relevant feedback points rather than feedback quality.

Overall, future work could aim to identify whether changes to the FC EPA assessment form or faculty development might improve technical feedback for trainees. Other work could determine how better to reduce assessment burden and continue to refine the role that EPA assessments play in trainee feedback more broadly.

## Conclusions

We showed that narrative comments from EPA intraoperative assessments completed through the FC contained relatively limited technical feedback of the kind included in the OSATS. While it is unreasonable to continue introducing additional assessment burden without eliminating existing assessments, our results suggest there may be a benefit to adjusting the FC EPA assessment form, further emphasizing faculty development, or continuing other types of targeted technical assessment intraoperatively.

## Funding sources

This was an unfunded project.

## Ethical approval

The University of California, San Francisco Institutional Review Board deemed this study to not require review (IRB22-38116).

## CRediT authorship contribution statement

**Riley Brian:** Conceptualization, Data curation, Formal analysis, Investigation, Methodology, Project administration, Resources, Software, Validation, Visualization, Writing – original draft, Writing – review & editing. **Natalie Rodriguez:** Conceptualization, Formal analysis, Investigation, Methodology, Writing – review & editing. **Connie J. Zhou:** Conceptualization, Formal analysis, Investigation, Writing – review & editing. **Megan Casey:** Conceptualization, Formal analysis, Investigation, Writing – review & editing. **Rosa V. Mora:** Conceptualization, Formal analysis, Investigation, Writing – review & editing. **Katherine Miclau:** Conceptualization, Formal analysis, Investigation, Writing – review & editing. **Vivian Kwok:** Formal analysis, Investigation, Writing – review & editing. **Liane S. Feldman:** Investigation, Methodology, Supervision, Writing – review & editing. **Adnan Alseidi:** Conceptualization, Investigation, Methodology, Project administration, Resources, Supervision, Validation, Writing – review & editing.

## Declaration of competing interest

The authors have no relevant financial or non-financial interests to disclose.
